# Successful Surgical Management of Incidentally Diagnosed Scimitar Syndrome With Anomalous Connection of the Left Hepatic Vein to Coronary Sinus and Persistent Left Superior Vena Cava

**DOI:** 10.7759/cureus.38367

**Published:** 2023-05-01

**Authors:** Rajeshwar Yadav, Aditya Sharma, Swati Pathak

**Affiliations:** 1 Cardiac/Thoracic/Vascular Surgery, Institute of Medical Sciences, Banaras Hindu University, Varanasi, IND; 2 General Surgery, Institute of Medical Sciences, Banaras Hindu University, Varanasi, IND

**Keywords:** scimitar syndrome, dilated coronary sinus, left hepatic vein, complex congenital heart disease, persistent left superior vena cava (plsvc), dextroposition of the heart

## Abstract

Scimitar syndrome is a rare congenital heart pathology that presents at birth, and it is a type of partial anomalous pulmonary venous return (PAPVR). In one in three people with scimitar syndrome, the right pulmonary vein is shaped like a scimitar (a Turkish sword) and can be easily visualized on radiographic imaging. This syndrome is not a simple and benign disease, and associated cardiac anomalies play a role in long-term outcomes, and the presence of pulmonary hypertension contributes to high mortality. The present case is the rarest of rare entities, scimitar syndrome, anomalous vascular connections between the left hepatic vein and coronary sinus, and persistent left superior vena cava in a single patient with no such case report published before.

## Introduction

Scimitar syndrome is a rare entity whose presentation ranges from asymptomatic to cardiac failure. Early presentation is usually associated with a congenital heart defect. In adults, it may be associated with hemoptysis, pulmonary hypertension, or cardiac failure [1]. Scimitar syndrome is a variant of a partial anomalous pulmonary venous connection resulting in a left-to-right shunt, also known as venolobar syndrome. The characteristic imaging feature resembles the Turkish sword "scimitar," hence the name. It is commonly associated with other congenital cardiac defects such as atrial septal defect (ASD) (20%-83%) [2]. The ventricular septal defect (VSD), patent ductus arteriosus (PDA), coarctation of the aorta, and persistent left superior vena cava (PLSVC) association have also been observed [3]. George Cooper first described it in 1836 while conducting an autopsy on a 10-month-old infant.

Imaging diagnosis (cardiac catheterization) was first made by Dotter et al. in 1949. Surgical intervention involving resection of the right lower lung was carried out for the first time in 1950 by Drake and Lynch. Corrective surgery was achieved for the first time in 1956 by Kirklin et al. [4]. This syndrome is a rare entity in and of itself; in this case, two additional unusual correlations are present. First, there is an incredibly rare congenital defect known as an anomalous connection of the left hepatic vein to the coronary sinus found incidentally during investigations or surgery [5]. It arises from the left hepatic lobe and passes through the diaphragm to join the body of the coronary sinus to the Ostia. Under tension or traction during surgery, it might cause arrhythmia and hypotension. The PLSVC is a thoracic venous anomaly and the most common cause of dilated coronary sinus, occurring in 3%-10% of patients with congenital heart disease [6]. It arises from the confluence of left internal jugular and subclavian veins, then courses ventral to the aortic arch, and pierces the pericardium to drain into the coronary sinus. Combining these three anomalies in a single patient makes it the rarest of rare cases, with no case reported yet. Hence, we report the first case with three congenital cardiac anomalies in a single patient.

## Case presentation

A 34-year-old housewife presented with chief complaints of breathlessness for three years, easy fatiguability for one year, and palpitations for two months. As stated by the patient, she was asymptomatic three years ago when she developed breathlessness, which had an insidious onset and was gradually progressive; it was aggravated by exertion and relieved by rest. It was also associated with generalized fatiguability and lethargy for the last year, initially due to exertion but gradually progressed to fatiguability even at less than ordinary daily activity. In the last two months, the patient developed palpitation, which was gradual in onset, usually occurred only on exertion, never at rest, and was associated with a forceful precordial activity. For the above complaints, the patient was investigated and diagnosed elsewhere with an ostium secundum atrial septal defect. The patient came to our cardiothoracic and vascular surgery outpatient department for further operative management of the then-diagnosed case of ostium secundum atrial septal defect.

There was no history of fever, cough with expectoration, or night sweats; joint pain associated with a throat infection; rashes; abnormal body movements; giddiness; syncope; convulsions; bluish discoloration of the skin, nails, or mucous membranes; squatting episodes; hematuria; skin lesions; blackening of fingers; dysphagia; or hoarseness of voice. There was no history of surgical intervention in the past. There were no associated comorbidities. She was married for 10 years, with two live issues and both deliveries uneventful. There was no history of similar illnesses in the family.

During the general examination, the patient was conscious, cooperative, and well-oriented to time, place, and person. On presentation, the pulse was 102 beats per minute, irregular, hypovolemic, and recorded in the right radial artery without any radio-radial or radio-femoral delay; the blood pressure was 100/66 mmHg (right brachial artery); the respiratory rate was 22 cycles per minute thoracoabdominal; she was averagely built and poorly nourished with a body mass index of 16.6. There was no icterus, clubbing, cyanosis, or generalized lymphadenopathy.

On systemic examination, the chest was bilaterally symmetrical, the apex beat was visible at the left sixth intercostal space, and there were no visible scar marks, sinuses, or dilated veins. During palpation, all the findings related to inspection were confirmed, and a diffuse apical impulse was felt at the left sixth intercostal space, just lateral to the midclavicular line. On percussion, the liver superiorly extended to the fifth intercostal space, and the left heart border corresponded to the apex.

On auscultation, a loud S1 was heard with a fixed split S2. A pansystolic murmur was heard at the tricuspid area, grade 2 at the lower parasternal region, which was high-pitched blowing and plateau in configuration, radiating to the apex, and best heard with the diaphragm. It increased on inspiration and the leg-raising test and decreased on expiration, suggesting tricuspid regurgitation.

An ejection systolic murmur of grade 2 is best heard at the left second intercostal space in the parasternal region, is short, crescendo-decrescendo early peaking, is of medium frequency, does not radiate to the carotid, and is best heard with the diaphragm at end-expiration in the pulmonary area (flow murmur). Moreover, findings may be summarized as situs solitus, levocardia, congenital acyanotic heart disease, unequal pulmonary flow, shunt physiology, a pre-tricuspid left-to-right shunt in the form of an atrial septal defect, atrial fibrillation, and New York Heart Association (NYHA) class 4 at present.

During the respiratory system examination, decreased air entry was noted on the right side, especially in the basal region, compared to the contralateral side. The abdomen was soft; mild tenderness was noted in the right hypochondrium, and mild hepatomegaly was present. The results of the central nervous system examination were within normal limits.

**Figure 1:**
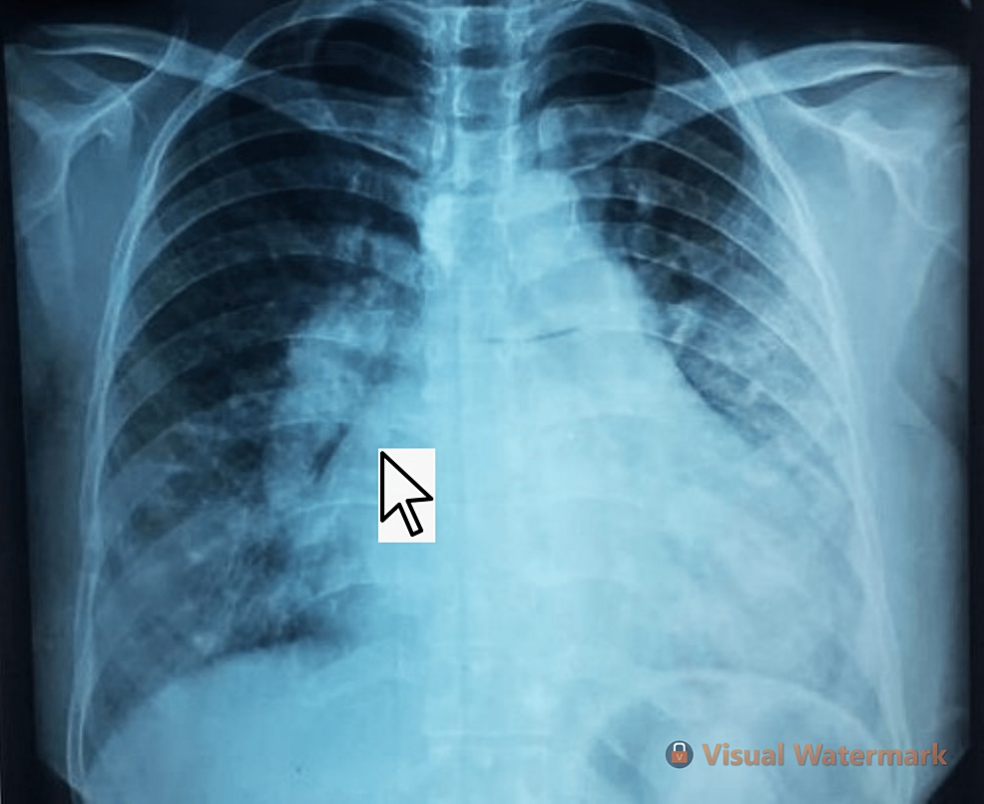
A chest X-ray posteroanterior (PA) view with a pointer illustrating the course of an anomalous vessel along the curvature of the right heart border and prominent pulmonary vasculature due to pulmonary artery hypertension

After the detailed clinical examination, the patient was subjected to a certain set of investigations. The chest X-ray revealed the anomalous vessel course, the curvature of the right heart border, and prominent pulmonary vasculature due to pulmonary artery hypertension as shown in Figure 1.

**Figure 2:**
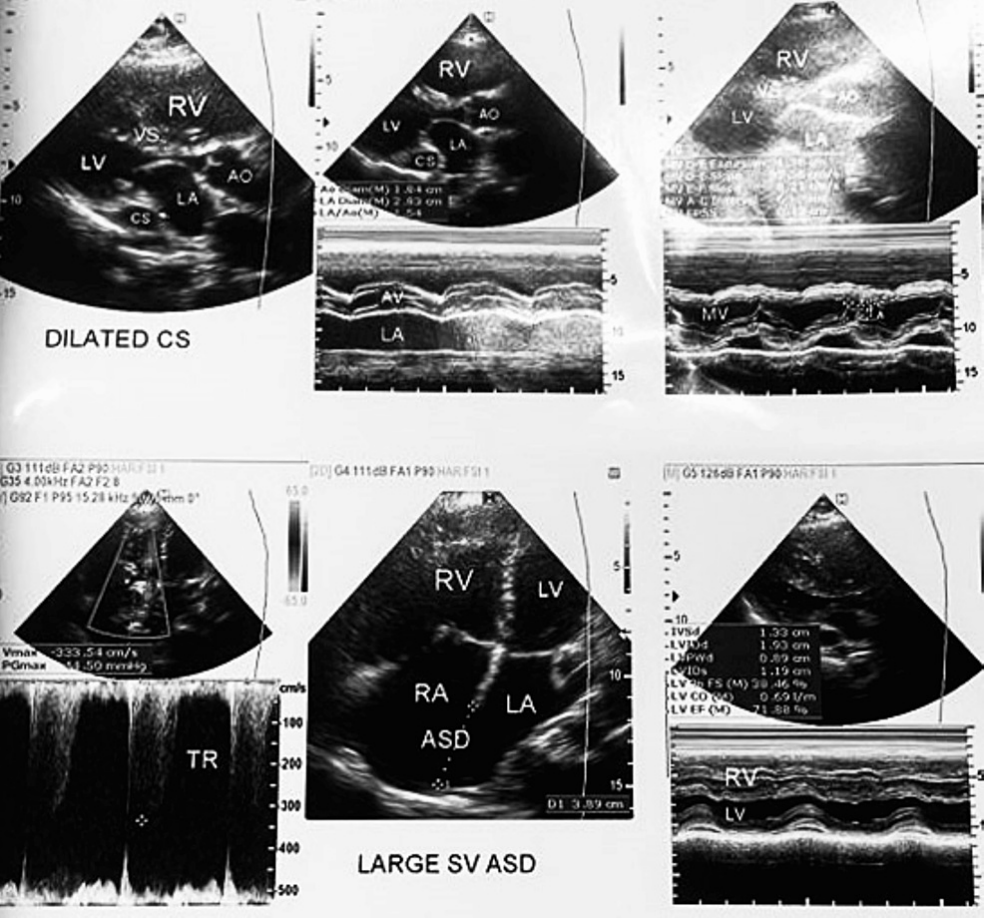
The transthoracic echocardiogram showing large sinus venosus atrial septal defect (superior vena cava type 40 mm), non-restrictive left-to-right arterial shunt, and pulmonary to systemic flow ratio (Qp/Qs) > 2.5:1 with marked right-side volume overload and preserved biventricular function

The transthoracic echocardiogram was in favor of acyanotic heart disease with large sinus venosus atrial septal defect (superior vena cava type 40 mm), non-restrictive left-to-right arterial shunt, and pulmonary to systemic flow ratio (Qp/Qs) > 2.5:1 with marked right-side volume overload and preserved biventricular function as shown in Figure 2.

Along with dilated main pulmonary artery, moderate pulmonary arterial hypertension, moderate tricuspid regurgitation, and dilated coronary sinus with persistent left hepatic vein draining into the coronary sinus are present. The right atrium, right ventricle, and right ventricular outflow tract are dilated, and the main pulmonary artery (29 mm) is markedly dilated. The left atrium, left ventricle, and the aorta appear small (underfilled). The interventricular septum is intact; motion is paradoxical. No PDA flow or aortic coarctation, moderate tricuspid regurgitation, and mild pulmonary regurgitation are seen, and pulmonary artery systolic pressure of 50 mmHg from the tricuspid regurgitation jet suggests moderate (hyperkinetic) pulmonary artery hypertension.

Other hematological investigations were within normal limits, like the complete blood count, liver function test, renal function test, and prothrombin time/international normalized ratio. Thus, on re-evaluation, the patient was diagnosed with a large sinus venosus atrial septal defect of superior vena cava type with a left-to-right atrial shunt. Dilated coronary sinus (PLSVC draining into the coronary sinus), moderate tricuspid regurgitation, and moderate pulmonary artery hypertension are present.

## Discussion

**Figure 3:**
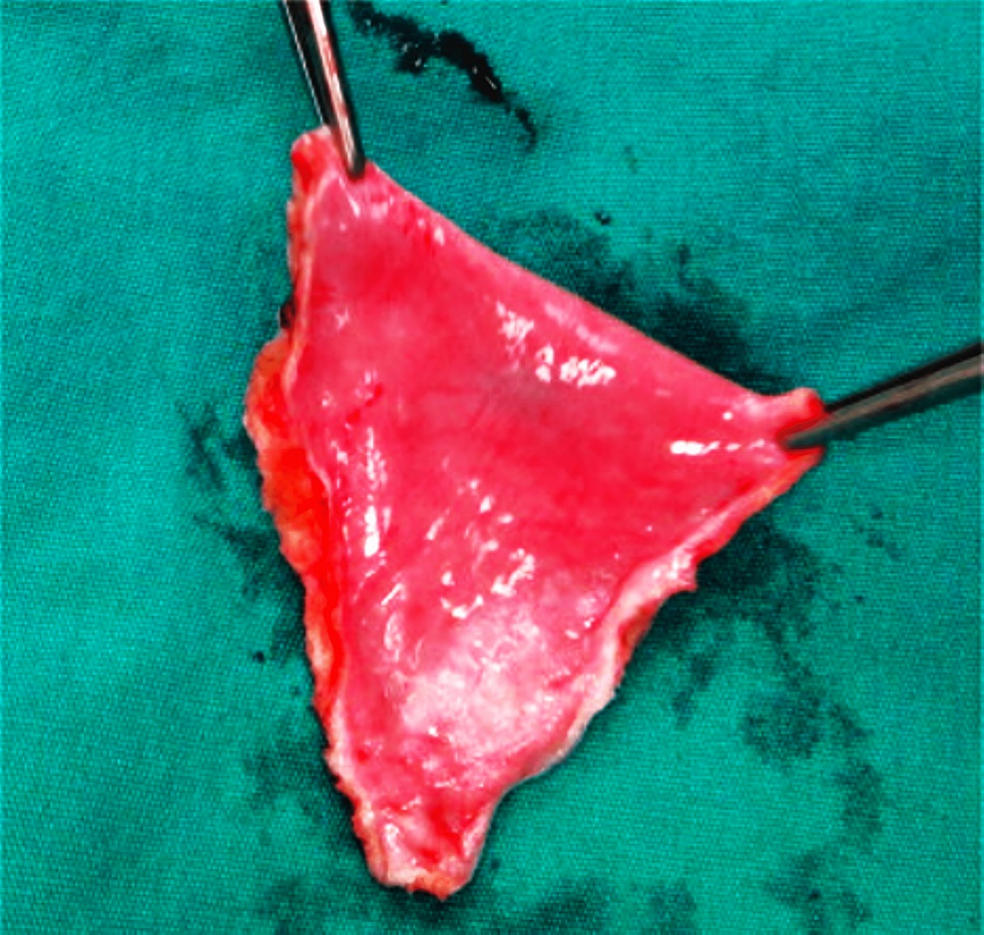
An autologous pericardial patch for the closure of the sinus venosus atrial septal defect

The patient was posted for elective surgery. Closure of the sinus venosus atrial septal defect via an autologous pericardial patch was planned as shown in Figure 3.

Central and peripheral venous lines, the radial arterial line, a temperature probe, and bispectral index monitoring were started on the table. Induction was achieved with 0.5 mcg/kg of fentanyl and 0.2 mg/kg of etomidate administered intravenously, 0.1 mg/kg of vecuronium-assisted tracheal intubation using a 7-mm inner diameter endotracheal tube. After the usual preparation and positioning of the patient, a median sternotomy was performed. The pericardium was cleared of pleural reflections, and a large pericardial piece was removed and secured. After taking the sutures, the anatomies were examined.

Operative findings were that the size of the superior vena cava was small due to the presence of the left superior vena cava. The right atrium and right ventricle were considerably enlarged. An anomalous vessel was visible running leftward and along the inferior vena cava within a distance of 1.5 cm and probably of infra-diaphragmatic origin (passing through the diaphragm). Surgery was carried out using the following operative steps: Purse-string sutures were placed for aortic and bicaval cannulations. A cardiopulmonary bypass was established, and the aorta was clamped. A cold cardioplegia was infused. The caval snares were secured. The perfusate temperature was stabilized at 30-degree centigrades to allow moderate hypothermia.

When the heart stopped, the right atrium was opened through the usual oblique incision, beginning at the base of the right atrial appendage and extending toward the inferior vena cava cannula. After the stay sutures were secured, a pump suction was introduced into the left atrium across the atrial septal defect. On examination of the interior of the right atrium, there was an enormously enlarged coronary sinus ostium. The left hepatic vein on the left of the inferior vena cava was also draining into the coronary sinus. A large sinus venous inferior vena cava of type atrial septal defect was present. One right superior and one right inferior pulmonary vein orifice were visible in the left atrium. An additional anomalous opening was also visible at the junction of the inferior vena cava and right atrium, almost inside the lumen of the inferior vena cava. This was an anomalous or accessorized right inferior pulmonary vein draining into the inferior vena cava close to the inferior vena cava-right atrial junction (scimitar vein).

To further repair, the perfusate was cooled and stabilized at 20 degrees. When deep hypothermia was achieved, circulatory arrest was established. The inferior vena cava cannula was temporarily removed, and under deep hypothermic circulatory arrest, the pericardial patch was sewn into place to form the anterior wall of a conduit between the entrance of the anomalous vein and the atrial septal defect. After the repair, the inferior vena cava cannula was reinserted, caval snares were retightened, the cardiopulmonary bypass was reestablished, and rewarming with the perfusate began. The right atrium was closed, and cardiopulmonary bypass was gradually discontinued as good ejections were established. Decannulation, reinforcement, and hemostasis were achieved. The sternum was closed, and the patient was shifted to the intensive care unit in stable condition.

Postoperatively, the patient was ventilated overnight to assist the slow and smooth adaptation of the heart and lungs to new hemodynamic flows. Minimal inotropic support was required. The patient did not have any cardiac rhythm events during the postoperative period. Moreover, treatment during the postoperative phase included adequate antibiotic coverage with good diuretic therapy; antiplatelet therapy was also begun. Tablet aspirin (75 mg) was given orally through Ryle's tube. Gradually, the mediastinal drain was removed by postoperative day three. The patient was ambulatory on postoperative day three and was discharged on oral medications on postoperative day 10 when her hospital stay was uneventful. After discharge, the patient was routinely followed, initially every 15 days, then every two months. As time passed, remodeling started magically reducing the cardiac size to normal values. Since anomalous lesions were present, follow-up required meticulous observation.

**Figure 4:**
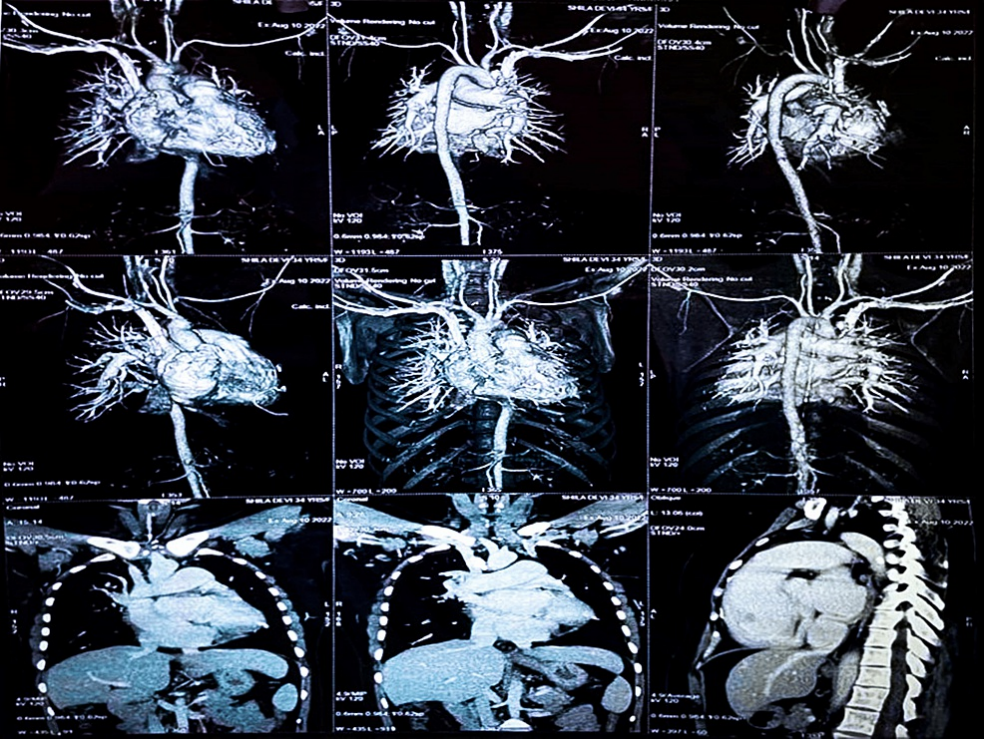
Follow-up CT angiography findings suggest mild cardiomegaly with relative enlargement of the right-side chambers and the left hepatic vein draining into the coronary sinus, along with persistent left superior vena cava draining into the coronary sinus

Follow-up computed tomography angiography findings suggest mild cardiomegaly with relative enlargement of the right-side chambers. The left hepatic vein drains into the coronary sinus, and the PLSVC drains into the coronary sinus as shown in Figure 4.

## Conclusions

This rare syndrome is present in 3%-6% of partial anomalous pulmonary venous connection cases. Variable presentations are seen, ranging from isolated lesions with benign outcomes to multiple associated lesions resulting in congestive cardiac failure or respiratory distress. There are two types of clinical manifestations: infantile and childhood/adulthood. The infantile type has a grave prognosis associated with multiple cardiac lesions and progresses to severe pulmonary arterial hypertension. These patients are diagnosed early (one year), and mortality ranges to about 45%. The childhood/adult variant manifests as frequent pulmonary infections and incidental findings of right-side heart dilatation due to mildly raised pulmonary artery pressures.

The present case is very rare and important as the left hepatic vein is an extremely rare congenital cardiac condition with only a published report. PLSVC is a surgical challenge since cannulation, and establishing cardiopulmonary bypass has become onerous. The patient was diagnosed with sinus venosus atrial septal defect, but the incidental finding of the anomalous vessel opening in the inferior vena cava was a surgeon's nightmare. Quick, witty decisions are important to reduce cross-clamp and cardioplegia times in cardiopulmonary bypass procedures. Swift resolution and preparation are required to go under deep hypothermic circulatory arrest. Teamwork involving the senior, anesthetist, and perfusionist is crucial when the patient is under deep hypothermic circulatory arrest. To successfully manage these patients, there is a need for the hour "Heart Team." A proper and thorough evaluation is necessary for such rare presentations before considering the operative correction. Precise knowledge of hemodynamics and apt decisions when incidentally landing up with numerous anomalous lesions are necessities for successful management. A deep hypothermic circulatory arrest is a double-edged sword and should be applied wisely.

## References

[1] Folger GM (1976). The scimitar syndrome. Anatomic, physiologic, developmental and therapeutic considerations. Angiology.

[2] Farnsworth AE, Ankeney JL (1974). The spectrum of the scimitar syndrome. J Thorac Cardiovasc Surg.

[3] Wang H, Kalfa D, Rosenbaum MS (2018). Scimitar syndrome in children and adults: natural history, outcomes, and risk analysis. Ann Thorac Surg.

[4] Aziz AA, Thomas S, Lautner D, Al Awad EH (2018). An unusual neonatal presentation of Scimitar syndrome. AJP Rep.

[5] Karolczak MA, Mądry W, Zacharska-Kokot E (2016). Anomalous connection of the left hepatic vein to coronary sinus in a child with PAPVD. Surgical significance and diagnostic difficulties. Kardiochir Torakochirurgia Pol.

[6] Anderson RC, Adams Jr P, Burke B (1961). Anomalous inferior vena cava with azygos continuation (infrahepatic interruption of the inferior vena cava). J Pediatrics.

